# Genome-wide transcriptome analysis of soybean primary root under varying water-deficit conditions

**DOI:** 10.1186/s12864-016-2378-y

**Published:** 2016-01-15

**Authors:** Li Song, Silvas Prince, Babu Valliyodan, Trupti Joshi, Joao V. Maldonado dos Santos, Jiaojiao Wang, Li Lin, Jinrong Wan, Yongqin Wang, Dong Xu, Henry T. Nguyen

**Affiliations:** Division of Plant Science and National Center for Soybean Biotechnology, University of Missouri, Columbia, MO 65211 USA; Department of Computer Science, and Christopher S Bond Life Sciences Center, University of Missouri, Columbia, MO 65211 USA; MU Informatics Institute, University of Missouri, Columbia, MO 65211 USA; Department of Molecular Microbiology and Immunology, School of Medicine, University of Missouri, Columbia, MO 65212 USA

**Keywords:** Soybean, Gene expression profile, Water-deficit, RNA-Seq, Hormone interplay, Metabolism, Transcription factor, Root

## Abstract

**Background:**

Soybean is a major crop that provides an important source of protein and oil to humans and animals, but its production can be dramatically decreased by the occurrence of drought stress. Soybeans can survive drought stress if there is a robust and deep root system at the early vegetative growth stage. However, little is known about the genome-wide molecular mechanisms contributing to soybean root system architecture. This study was performed to gain knowledge on transcriptome changes and related molecular mechanisms contributing to soybean root development under water limited conditions.

**Results:**

The soybean Williams 82 genotype was subjected to very mild stress (VMS), mild stress (MS) and severe stress (SS) conditions, as well as recovery from the severe stress after re-watering (SR). In total, 6,609 genes in the roots showed differential expression patterns in response to different water-deficit stress levels. Genes involved in hormone (Auxin/Ethylene), carbohydrate, and cell wall-related metabolism (XTH/lipid/flavonoids/lignin) pathways were differentially regulated in the soybean root system. Several transcription factors (TFs) regulating root growth and responses under varying water-deficit conditions were identified and the expression patterns of six TFs were found to be common across the stress levels. Further analysis on the whole plant level led to the finding of tissue-specific or water-deficit levels specific regulation of transcription factors. Analysis of the over-represented motif of different gene groups revealed several new cis-elements associated with different levels of water deficit. The expression patterns of 18 genes were confirmed byquantitative reverse transcription polymerase chain reaction method and demonstrated the accuracy and effectiveness of RNA-Seq.

**Conclusions:**

The primary root specific transcriptome in soybean can enable a better understanding of the root response to water deficit conditions. The genes detected in root tissues that were associated with key hormones, carbohydrates, and cell wall-related metabolism could play a vital role in achieving drought tolerance and could be promising candidates for future functional characterization. TFs involved in the soybean root and at the whole plant level could be used for future network analysis between TFs and cis-elements. All of these findings will be helpful in elucidating the molecular mechanisms associated with water stress responses in soybean roots.

**Electronic supplementary material:**

The online version of this article (doi:10.1186/s12864-016-2378-y) contains supplementary material, which is available to authorized users.

## Background

Soybean is the third most cultivated crop worldwide and the most important vegetable oil and protein source for humans. Soybean production is limited by environmental constraints, particularly drought [1, 2]. Water availability to the plant is the most important factor, as soybeans use approximately 450–700 mm of water during the growing season [3].

Deeper root growth is important for maintaining crop yields, especially under drought stress conditions. It has been reported that low water potential at soybean vegetative stages will decrease or stop its shoot growth, while the root continues to grow [4]. This shoot-root response in soybean under water-deficit conditions allows the plant to search for additional soil water while maintaining higher water use efficiency [5]. However, the limited vegetative plant growth and reduced levels of photosynthesis under drought stress negatively affect the soybean yield potential. In soybeans, short-term and moderate drought stress during vegetative growth stages generally does not impact the soybean yield in comparison with the longer-term and severe drought stress. Soybean genotypes with the ability to develop a larger root system before the occurrence of drought stress at the flowering stage were reported to have higher yield performance [6]. Root growth was less affected when drought was imposed at the reproductive R4 stage and ceased at the R5 stage [7]. The taproot elongation rate is the major factor influencing the depth of the soybean rooting system under water-deficit conditions [8]. These results indicated that the lack of soil moisture at critical stages of growth will profoundly impact the productivity and that developing a large root system at the early vegetative (V) growth stage will place the soybeans in an excellent position to maintain turgor under drought conditions.

Therefore, understanding the soybean taproot response to drought is very critical for effective management of abiotic stress. Consequently, understanding the genetic regulation of the taproot response to drought will help to identify specific genes and metabolic pathways for either gene-based marker selection or genetic engineering to develop soybeans with better root-related traits. Accordingly, characterization of water-deficit induced changes in transcripts within the growth zone of the primary root is important to understand the mechanisms that control the response of root growth to water deficits. Until now, the molecular events associated with drought stress in soybean taproots are not well known and few studies have analyzed differences in transcriptional responses to different drought conditions in the same tissue or whole plant. Investigation and understanding of the responsible genomic mechanisms for drought stress will be useful to improve varieties with yield protection in soybean breeding programs. The novel genes involved in root development under drought conditions through expression pattern analysis can also be used for further functional genomic studies.

With the advent of next-generation sequencing (NGS) methods, Illumina/Solexa’s sequencing technology has been used for understanding the complexity of gene expression and regulation networks in several plant species responding to abiotic stresses, including cotton [9], Chinese cabbage [10], *chickpea* [11], maize [12], *Ammopiptanthus mongolicus* [13], and soybean [14]. The research in soybean response to drought was investigated in soybean slow-wilting (PI 416937, PI 471938 and PI 567690) and fast-wilting lines (Benning, Hutcheson and Pana) [15–17]. Both genotypic and non-genotypic differential genes response to water deficit were identified, which extends the current understanding of plant hydraulic conductivity. Several high potential candidate genes were also identified to elucidate the mechanism of slow-wilting. However, all of these experiments used leaf tissue to profile the gene expression pattern under water-deficit or high vapor pressure deficit (VPD) conditions. Recently, the root transcriptomes of DT2008 and W82 soybean seedlings under short-term (2 h or 10 h) dehydration conditions were analyzed by use 66 K Affymetrix Soybean Array GeneChip [18]. The differential expression of genes (osmoprotectant biosynthesis, detoxification or cell wall-related proteins, kinases, transcription factors and phosphatase 2C proteins) may cause higher drought tolerability of DT2008 vs. W82. This research enable us to identify early responsive genes and understand the upstream regulation mechanism of soybean root response to dehydration. However, no research has focused on how the soybean taproot responses to different drought level treatments until now.

To provide novel insights into the molecular basis of drought stress in the soybean root, genome-wide transcriptome profiling using Illumina/Solexa’s sequencing approach has been used at different drought conditions in the present work. First, the comparative analysis in the root between well-watered and drought stress conditions showed several key metabolic and hormone pathways involved in regulating the root response under varying water deficit levels. Secondly, the TF analysis led to the identification of key molecular regulators to improve drought tolerance/avoidance. These findings will benefit the elucidation of mechanisms for temporal and spatial regulation of genes in soybean under drought conditions.

## Results

### Phenotypic response of the soybean shoot and leaf to varying water deficit treatments

**Fig. 1:**
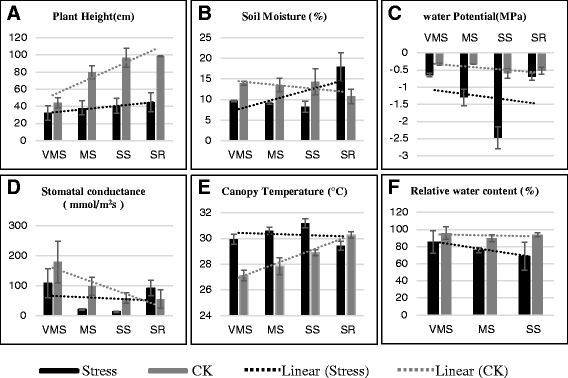
Soybean (Williams 82 genotype) phenotypic responses to different water-deficit treatments. Soybean plant height (**a**), soil moisture (**b**), leaf water potential (**c**), leaf stomatal conductance (**d**), leaf canopy temperature (**e**), and leaf relative water content (**f**) were monitored to indicate the water-deficit levels. CK (well-watered control), VMS (Very Mild Stress), MS (Mild Stress), SS (Severe Stress), SR (water Recovery after severe stress). Bars indicate standard error of the mean of nine plants

The phenotypic response of the soybean shoot and leaf was monitored to determine the water deficit levels. The reduction of plant height was first observed under a very mild water deficit stress treatment (VMS). The height of the plants under a severe stress (SS) treatment was nearly half compared to the plant height in the well-watered treatment (Fig. 1a). The level of water deficit imposed was evident from the depletion of soil moisture. Compared with the well-watered treatment (13.9 %), after 5 days of no water, the soil moisture percentage decreased under very mild stress to 9.7, mild stress to 9.1 and severe stress to 8.3. The soil moisture increased to 17.9 after water recovery (Fig. 1b). The plant water status was also revealed by the leaf water potential (LWP) values in drought stressed pots (Fig. 1c). The leaf water potential reached in this experiment reflected the severe drought stress treatment in the field with a LWP of −2.5 MPa (megapascals). During the stress recovery treatment, the severely stressed pots were re-watered for two days to recover, reaching a water potential of −0.65 MPa. In addition to the general decrease in growth caused by the water deficit, several physiological indicators were monitored under different water deficit stages. The plants under the drought stress treatment showed declined stomatal conductance values up to 80–90 % in the MS and SS stress treatments, compared to the VMS treatment (Fig. 1d). This in turn affected the canopy temperature (Fig. 1e). The leaf relative water content (RWC) was also found to decrease with the varying water deficit levels from 85.2 % (VMS) to 68.6 % (SS) (Fig. 1f). The effect of different stress levels is evident in the soybean plants with varying physiological responses. In the water recovery treatment, the leaf water potential and stomatal conductance values of the plants were the same as observed in the VMS treatment.

### Identification of differentially expressed genes (DEGs) under different water-deficit stresses in the soybean taproot

**Fig. 2:**
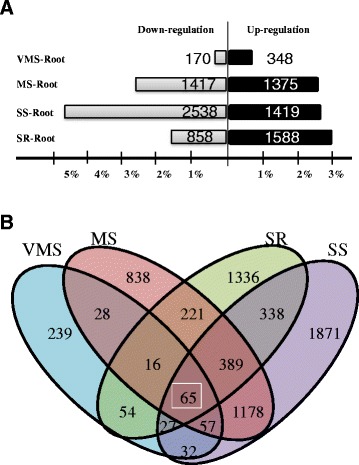
Distribution and Venn diagram display of differentially expressed genes (DEGs) in soybean root responses to different water-deficit treatments. **a** Number of DEGs under different water-deficit treatments in the root. (R value < 0.05 and genes with the regulation ratio log2 ≥ 2 or ≤ -2 were selected) Gray: down-regulated. Black: Up-regulated. **b** Venn diagram showing the overlapping number of DEGs under different water-deficit treatments in the root (Blue: VMS, Pink: MS, Green: SR, Purple: SS)

To judge the significance of gene expression differences, the following criteria were used: R value < 0.05, and │log2│ ratio ≥ 2. In the varying stress levels and stress recovery treatments, different numbers of genes were found to be differentially regulated in taproot tissue: VMS (518 genes), MS (2,792 genes) and SS (3,957 genes) and SR (2,446 genes). In total, 6,609 genes showed significantly changed expression patterns under at least one stress treatment. As shown in Fig. 2a, fewer genes were differentially expressed in the VMS treatment and more genes were down-regulated in the SS treatment than the MS and SR treatments. However, more differentially expressed genes exhibited up-regulation after water recovery compared to the SS treatment. The Venn diagram analysis of different levels of water-deficit treatments in the root tissue showed higher numbers of common genes detected between MS and SS stresses. SR shared more genes in common with SS than VMS and MS treatments. In total, 65 genes were found to show centered changes at all different stress levels (Fig. 2b).

**Fig. 3:**
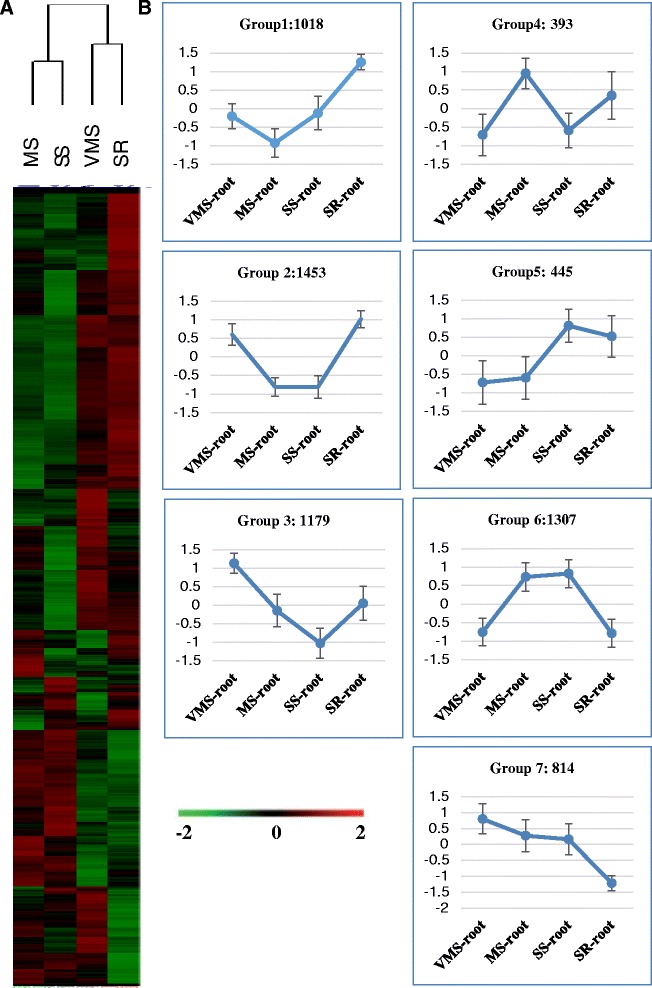
Expression profile of differentially expressed genes (DEGs) clustering. **a** Hierarchical clustering display of the up- and down-regulated genes in the root indicated the gene expression patterns of severe stress were more similar to mild drought stress. Very mild stress and recovery showed a correlation. **b** Root gene expression profile clustering showed seven groups with normalization values between 2 and -2. Standardized expression levels are shown on y-axis and stress level on x-axis. Each group is represented by the gene expression profiles under different drought levels. Group1: genes up-regulated under SR treatment, but down-regulated under MS treatment, constantly under VMS and SS treatments; Group2: genes up-regulated under VMS and SR treatments, but down-regulated under MS and SS treatments; Group 3: genes up-regulated under VMS treatment, but downregulated under SS treatment, constantly under MS and SR treatments; Group4: genes up-regulated under MS treatment, but down-regulated under VMS and SS treatments, constantly under SR treatment. Group 5: genes up-regulated under SS and SR treatments, but down-regulated under VMS and MS treatments; Group6: genes up-regulated under MS and SS treatments, but down-regulated under VMS and SR treatments. Group 7: genes up-regulated under VMS treatment, but down-regulated under SR treatments, constantly under MS and SS treatments

Cluster analysis of the 6,609 genes with dChiP software indicated that the regulated genes under SS showed higher similarity with MS and VMS exhibited a higher correlation with SR (Fig. 3a). The clustered genes were further divided into seven subgroups to identify their connection with different pathways (Fig. 3b). The genes in Group 2 showed up-regulation in VMS, down-regulation in MS and SS, and the expression pattern in SR was very similar to that of VMS. However, the genes in Group 6 showed the opposite regulation pattern (Fig. 3b).

### Transcripts associated with metabolism were down-regulated in soybean roots under mild and severe water-deficit treatments

**Fig. 4:**
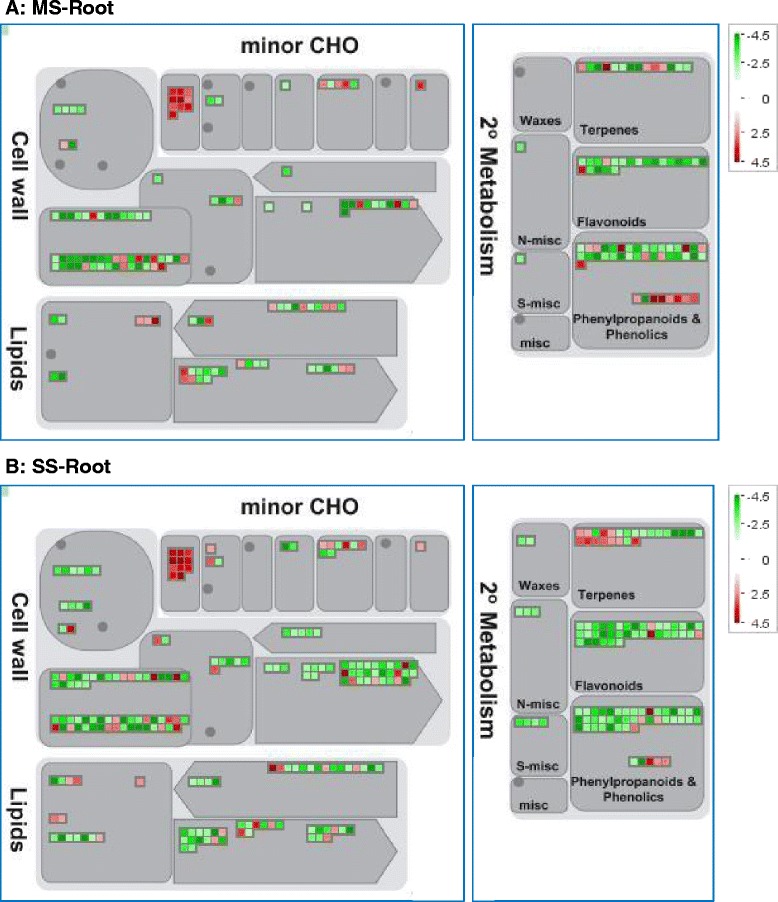
MAPMAN visualization of metabolism-related gene expression in water-deficit stressed soybean roots compared with control. The log2 fold changes of significantly DEGs were imported and visualized in MapMan for the root samples under MS condition (**a**) and SS condition (**b**). Genes were assigned to their associated metabolic pathways. Each square corresponds to a gene. Red squares indicate genes suppressed in comparison to isolated or untreated plants. Green squares indicate genes induced. MapMan version 3.6.0 was used to generate these images from gene expression data. The scale bar is shown in log2 from -4.5 to 4.5

Multiple metabolic related pathways that respond to water-deficit treatments have been identified. Transcripts associated with cell wall modification, such as expansins, xyloglucan endotransglucosylase/hydrolases (XTHs), and pectin methylesterases (PMEs), were identified in the root tissues. The transcripts within these three gene families were down-regulated during MS and SS treatments. Several gene families (mannan-xylose-arabinose-fucose, pectate lyases, and polygalacturonases) involved in cell wall degradation were mainly down-regulated (Fig. 4a and b and Additional file 1). Most of the differentially expressed genes involved in lipid metabolism (including fatty acid (FA) synthesis, elongation, desaturation, and degradation) were inhibited under MS and SS, which could limit lipid biosynthesis. None of the amino acid metabolic pathway genes was found to be differentially expressed in roots, but some related genes were found to be down-regulated in shoot tissues under drought stress (data not shown). Several gene families involved in the secondary metabolism pathways were found to be down-regulated under the MS and SS treatments, but most of them returned to normal level after water re-application. These include flavonoids, isoprenoids, lignin, fermentation, and glycolysis related genes (Fig. 4a and b and Additional file 2).

All of these results together demonstrated that water stress has negative effects on the metabolic processes of soybean roots and the down-regulated metabolism may slow down root growth in mild and severe drought treatments.

### Carbohydrate metabolism in soybean roots under water-deficit stress

It is known that carbohydrates accumulate in plants as a response to drought [19], but the alteration in metabolites and pathways specific to taproot responses still remain unknown. Differential expression of genes involved in sucrose and starch metabolism after imposition of water-deficit stress were found in the current study.

**Fig. 5:**
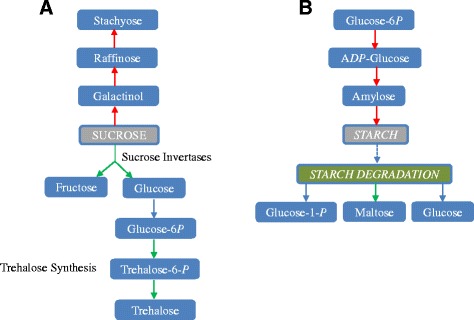
The regulations of carbohydrate metabolism in soybean roots under water-deficit stress. The transcripts associated with starch synthesis increased (**a**) and the transcripts associated with starch decreased (**b**) in MS and SS treatments. Red arrow: up-regulated; Green arrow: down-regulated. Blue arrow: no changes

Many genes encoding sucrose invertases that are present in the cell wall and vacuoles were decreased at a transcript level and the genes associated with trehalose synthesis were also down-regulated. Moreover, most of the transcripts showed a significant reduction in the SS treatment, which sends the oxidative stress signal to the plant. Conversely, galactinol and raffinose synthase related genes were up-regulated, which might act as an energy source to maintain root growth in drought stress (Fig. 5a, Additional file 3). No sucrose biosynthesis genes were found to change in the primary root during drought stress. Several starch branching and synthase enzyme related genes were slightly down-regulated in VMS, but were increased under MS and SS treatments. At the same time, the enzymes involved in starch degradation (starch cleavage) were also down-regulated (Fig. 5b, Additional file 3). Overall, there was an increase in transcripts associated with starch synthesis and a decrease in transcripts associated with starch degradation in MS and SS treatments. These results indicated that water-deficit induced transcripts change with carbohydrate metabolism, which in turn regulates sucrose and starch content. The increased content in galactinol and raffinose will enhance osmotic tolerance in the root under drought stress.

### The hormone regulation network under mild and severe water-deficit treatments in soybean roots

Hormones play a central role in integrating the environmental and intrinsic cues during plants adapt and grow under suboptimal treatments [20]. To elucidate the key hormones associated with root development under water-deficits, different hormone regulations of transcripts were investigated in this study. In total, 37 transcripts associated with auxin synthesis and signaling pathway genes were found to be regulated by drought. Among them, genes related to the auxin biosynthesis pathway (IAA-leucine resistant-like gene 66, indole-3-acetic acid-amido synthetase3.1 and 3.6, IAA-amino acid hydrolase IAR3) were found to be down-regulated upon water-deficit and showed up-regulation after re-watering. Concurrently, two auxin transporter genes (PIN2 and PIN5) that were down-regulated during drought treatments, exhibited an increase in the transcript levels during recovery. Several auxin responsive genes were detected to be regulated differently at varying levels of drought stress (Additional file 4). These results identified several key auxin signal and transporter genes that were regulated by water-deficit in the taproot.

It has been reported that the effect of water-deficiency on ethylene synthesis was linked with the plant’s rate of stressed [21, 22]. Rapid induction of water-deficit stress induces ethylene production and a slower imposition of water-deficit stress inhibits ethylene biosynthesis [21, 22]. In our results, 102 transcripts associated with the ethylene pathway were regulated, and most of the ethylene signal transduction and synthesis related genes were down-regulated in MS and SS treatments, but resumed at the recovery phase. They were mainly grouped into subgroup1, subgroup2 and subgroup3 (genes up-regulated or constantly under VMS and SR, down-regulated or constantly under MS and SS, Additional file 5). The ACC oxidases, ACO1 and ACO4, which catalyze the last step of the ethylene biosynthesis pathway, were found to be inhibited by water-deficit stress. Several ethylene response factors (ERFs) and ACC synthase (ACS) genes in ethylene pathway were also found to be down-regulated (Additional file 5). These results suggested the stress treatment in this study was a slow progression of drought induction. Ethylene synthesis and signal transduction played an important role in the soybean primary root growth under the water stress treatments.

Genes potentially involved in other hormonal pathways were also identified in the present study: abscisic acid (ABA, 22 genes), gibberellin (GA, 25 genes), jasmonic acid (JA, nine genes), salicylic acid (SA, nine genes) and cytokinin (16 genes) (Additional files 6 and 7). No brassinosteroid related genes were found. Although ABA is the major hormone known to be involved in drought stress response, there were only a few ABA related genes that changed in expression level under water deficient treatments. Among them, the transcripts of ABA synthesis genes,*NCED* (9-cis-epoxycarotenoid dioxygenase) and *CCD8* (carotenoid cleavage dioxygenases), increased with respect to the level of drought imposed. This trend was found to be reversed after water recovery. Several genes involved in the crosstalk between the two hormones were also found here. Two GA synthesis related genes, *GA2OX1* and *GA2OX2* (gibberellin 2-oxidase), were found to be down-regulated, which were also involved in the ethylene pathway. CCD7 and CCD8 (carotenoid cleavage dioxygenases), which function in root branching by involving ABA and the strigolactones synthesis pathway, were shown to change expression patterns under water-deficit treatments. Another gene, *GmJAR1* (JASMONATE RESISTANT 1), which is involved in both the auxin and JA pathways, was also inhibited during the drought stress treatment and recovered after re-watering. All of the above results indicated that the ethylene and auxin synthesis and signal transduction play a more important role than other hormones in the soybean taproot response to water deficient stress. On the other hand, hormone crosstalk probably plays a significant role in soybean root responses to drought.

### Transcription factor (TF) modulation network in the soybean root

**Fig. 6:**
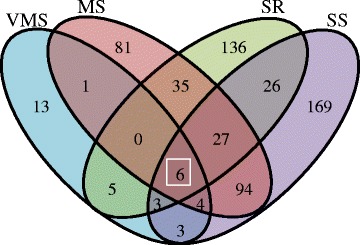
Venn diagram showing the number of transcription factors expressed in very mild stress (VMS), mild stress (MS), severe stress (SS), and water recovery after severe stress (SR) treatments in the root tissue. (Blue: VMS, Pink: MS, Green: SR, Purple: SS)

In total, 603 TFs exhibited altered expression patterns in the soybean taproots under varying levels of water-deficit treatments. Among them, 35 TFs were identified in VMS treatment, 248 TFs in MS treatment, 332 TFs in SS treatment and 238 TFs in SR treatment (Additional file 8). TFs accounted for between 6.75 and 9.8 % of the total number of differentially expressed genes in this study. A Venn diagram illustrating the classification of TFs into different groups based on varying water-deficit levels is shown in Fig. 6. Only a few TFs were regulated during VMS, but more specific TFs encoding transcripts were regulated in MS, SS, and the recovery phase, with the highest transcripts regulated in the SS treatment. The MS and SS may have similar regulatory and response mechanisms as they share more common TFs. Conversely, less common TFs between SS and SR indicated that the soybean root went through a quite different process after re-watering.

The TFs specifically regulated in both MS and SS treatment were selected for further gene family analysis (Additional file 9). Genes belonging to the bHLH (basic helix-loop-helix), AP2 (activating Protein 2), MYB (myeloblastosis) and WRKY (is a class of DNA-binding proteins that recognize the TTGAC(C/T) W-box elements) families represent most of the differentially expressed TFs. MYB represents the highest number of transcripts under drought followed by WRKY and bHLH. Six TFs were found to change under every stress level and they probably play the conserved or fundamental roles in taproot responses to stress. One of them, a heat shock related TF (Glyma04g05500), was up-regulated in all stress levels. Its homologue was found to regulate the heat stress response in Arabidopsis [23]. Another ERF (Ethylene response factor, containing the AP2 domain) TF was down-regulated. Two MYB15 TFs and one U-box TF were found to be up-regulated under VMS and SR treatments and were down-regulated under MS and SS treatments. In Arabidopsis, the AtMYB15 gene was found to be involved in the ABA, ethylene, and jasmonic acid mediated signaling pathways. Overexpression of AtMYB15 showed an improved survival rate and reduced water loss under water deficit treatments [24].

### Regulation of TFs in soybean whole plant responses under drought stress

The whole plant acts as a system to respond differently to stress treatments with spatio-temporal transcript regulation. Transcriptional control of stress-response gene expression is a crucial component of plant responses to a range of varying levels of environmental stresses. Understanding the regulation of TFs at a whole plant level during water deficit treatments will enable identification of key master regulatory elements involved in plant responses. The regulatory mechanisms of drought responses were specifically identified in the root water-deficit response and their regulation patterns were also evaluated in leaf and shoot tissue transcriptomes.

**Fig. 7:**
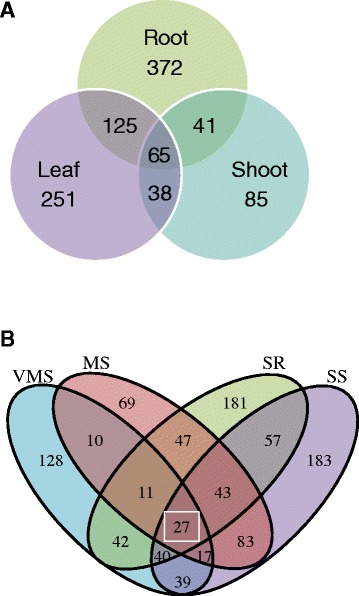
Comparative analysis with transcription factors (TFs) revealed the number of overlapping TFs from those three tissues or water-deficit treatment levels. **a** Venn diagram showing the TF number of DEGs under all water-deficit treatments in the different tissues (root, leaf, and shoot). **b** Venn diagram showing the transcription factor number of DEGs under different water-deficit treatments in three tissues (root, leaf, and shoot). (Blue: VMS, Pink: MS, Green: SR, Purple: SS)

First, to specifically uncover the regulatory mechanisms of drought responses in root tissue, all of the TFs in the DEGs were identified from the leaf and shoot tissues. As shown in Fig. 7a, the maximum number (603 TFs) of TFs were found in roots, with more than half of them (372 TFs) being root specific. A fewer number of TFs (229 TFs) encoding transcripts were found in the shoot in comparison to root and leaf transcriptomes. In addition, more than half of the TFs in the shoot were shared in the root and leaf as well. Sixty-five TFs were found in all three tissues (Additional file 8 and Fig. 7a). These results indicated that the gene regulation network between root and leaf plays more important roles under water-deficit treatments. Second, based on stress level specificity from the whole plant expression pattern, the TFs identified in the present study were classified. Minimum numbers (69 TFs) of stress level specific TFs were found in MS treatment, which also indicated that the mild stress level share more common TFs among other stress levels. Only a few specific TFs involved in the VMS stress were found in the root, but more were found at the whole plant level. This indicates more TFs were regulated in the shoot and leaf as an early drought stress response. Nearly half of the TFs regulated by SS or SR treatment were not adjusted by other stress level. Irrespective of the tissues used, 27 TFs found were involved in all of the stress treatments (Additional file 8 and Fig. 7b). Finally, 13 TFs were found to be regulated in all three tissues under all stress levels. Among them, five AP2 TFs were found to be highly induced under MS and SS treatments in the root and SR in the leaf tissue, and they were down-regulated in MS and SS treatments in the shoot tissues. UBIQUITINATION FACTOR E4 (Glyma.03G202600) and bHLH (Glyma.15G170500) TFs were decreased in number under MS and SS in all tissues studied. These results indicated that these common TFs are likely to play a key role in regulating the whole plant response under varying levels of drought stress, but might also be involved spatio-temporal regulation among different tissues (Additional file 10).

### Conserved motif analysis in the promoters of regulated genes in the soybean root

The promoter motifs, which are conserved in clusters of co-expressed and functionally related genes, may be involved in mediating coordinated gene activity [25, 26]. Over-represented sequences analyses were conducted on the promoter regions (2,000 base pairs upstream of the start codon) and all of the enriched motifs from the different drought response groups are listed in Additional file 11.

In this analysis, two motifs (AAAAAAAA and TATATATA) over-represented in each group were identified. The second motif was normally found approximately 25–35 base pairs upstream of the start site. Several known motifs involved in the known drought response pathway or BR pathway were also identified, such as CRTGCAY (ABI3), GCCRCS (ERF1), CACRC and ACGHGK (BES1), CTCYCYC (ABI4), and GMCACGB (ABF1, BZR1, and BES1). This result indicated that those enriched motifs were probably involved in the drought regulations network induced at different stress levels.

Because groups 2 and 6 (group2: genes up-regulated under VMS and SR treatments, but down-regulated under MS and SS treatments; group6: genes up-regulated under MS and SS treatments, but down-regulated under VMS and SR treatments;) stand for oppositely regulated mechanisms in response to drought in the taproot tissues, motif analysis on genes from these two groups in the root tissues were further assessed. Fifty-seven over-represented motifs were identified in the MS and SS up-regulated drought response genes group (Group 2) and 49 over-represented motifs were isolated in the MS and SS down-regulated drought response genes group (Group 6). Only one motif (CACGHG) overlapping in these two groups. The CACGHG motif was a “G-box”, which is the binding site of the GBF4 TF in Arabidopsis. It was also conserved in the PIF, BES ((BRI1-EMS-SUPPRESSOR 1), and MYC TF target element. Enriched sequences regulated by ARR10, ANT, and Dof3 TFs were found only in Group 2 and not in Group 6. The ARR10 TF is involve in the cytokinin signaling pathway and often shows a down-regulated response to abiotic stress [27]. *ANT* encodes an AP2-like ethylene-responsive TF and its function is most closely related to floral development [28]. It has been reported that the expression pattern of the DOF TF responds to abiotic stress in several species [29, 30]. Two enriched cis-elements (CWCCACS and CAYSCAC) were found in Group 6, which were regulated by TF GAMYB. It has been reported that GmGBP1, which is one soybean GAMYB binding protein, regulates flowering and improves drought tolerance in Arabidopsis [31]. These results indicated that different enriched motifs were involved in very specific regulation mechanism responses to different drought levels.

### qRT-PCR (Real-time quantitative PCR) validation of differentially expressed transcripts from RNA-Seq

To validate the RNA-Seq expression data and its reliability, 18 DEGs were selected for qRT-PCR analysis. To compare these two different methods, the relative expression measurement from the qRT-PCR was transformed into fold change by base 2 to match with the RNA-Seq fold change value.

**Fig. 8:**
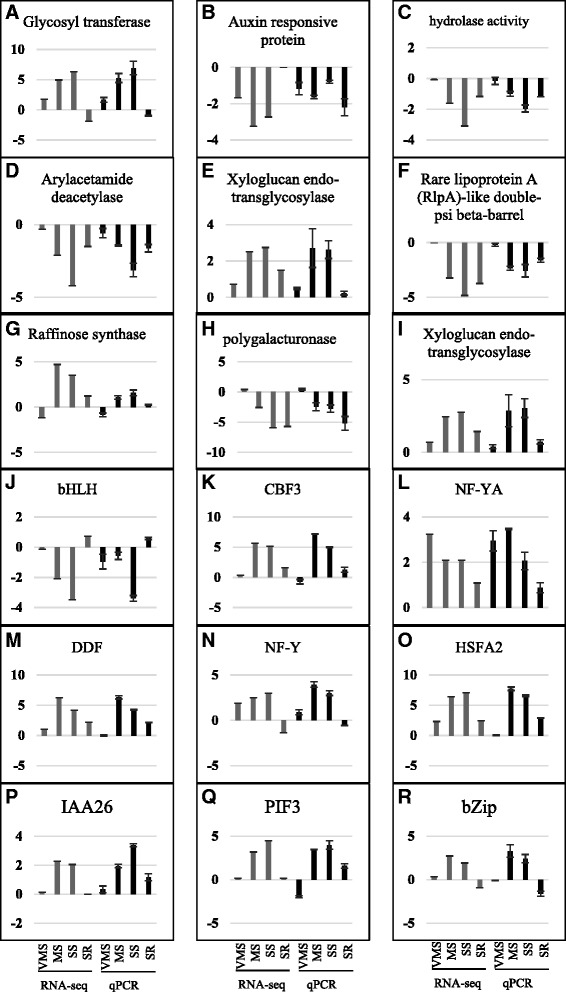
qRT-PCR analysis of genes identified to be water-deficit responsive by RNA-seq. Nine selected metabolism (**a-i**) and nine transcription factor (**j-r**) related DEGs with different drought stress treatments compared with the RNA-Seq data. Gray and black bars for RNA-Seq and qRT-PCR results, respectively. Error bars represent the Standard error for three independent experiments and two technical replicates

The 18 selected genes for this comparison included nine genes associated with metabolism and nine genes encoding TFs. As shown in Fig. 8(a–i), two cell wall-related genes (xyloglucan endo-transglycosylase) and two carbohydrate metabolism related genes (glycosyl transferase and raffinose synthase) showed up-regulation under MS and SS treatments. In addition, five genes (auxin responsive protein, hydrolase activity, arylacetamide deacetylase, rare lipoprotein A, and polygalacturonase) were significantly down-regulated under MS and SS treatments, and the gene expression never returned back to normal treatments at VMS and water recovery phases. Among the TFs selected, bHLH TFs showed a reduced expression pattern in all stress levels with increased transcripts during the recovery phase (Fig. 8j). However, the expression pattern of NY-YA was highly induced at all stress levels (Fig. 8l). All other transcription factors were significantly induced only under the MS and SS treatments in the RNA-Seq results (Fig. 8k–r).

After comparing the results between qRT-PCR and RNA-Seq, the expression patterns from the qRT-PCR were highly consistent with the RNA-Seq results. Furthermore, a correlation between RNA-Seq and qRT-PCR was evaluated using log2 expression levels. As shown in Additional file 12, the qRT-PCR measurements were highly correlated with the RNA-Seq results (Y = 0.8476x + 0.2348, R^2^ = 0.8346). The comparison of data between different methods showed the accuracy of the RNA-Seq method and showed that our RNA-Seq data were accurate and efficient. The RNA-seq results can be used for gene expression profiling during the soybean root response to water-deficit stress. Notably, a few genes showed different expression patterns between the RNA-seq and qRT-PCR data. Such inconsistency was probably caused by the relatively low expression of these genes and by no significant changes being found in the RNA-seq results.

## Discussion

The right root system architecture will allow plants to survive from a water deficit environment with an increasingly deeper, more vertically orientated root system to access the deep soil water. Spollen et al. reported that the elongation rates of soybean primary roots were less sensitive than the shoot and the growth of the shoot had more inhibition than the root under the same water potential [32]. Our phenotypic data from the shoot indicated that there is a good correlation between root water uptake capacity and shoot dry weight under water-limited conditions. This study unraveled the signaling networks, including hormone signaling, metabolism, and TFs in soybean primary root responses to the varying water-deficit conditions. Exploring the complex crosstalk between different regulatory levels involved in the soybean stress response will promote understanding of the regulation network of drought stress.

### Metabolic changes in soybean root under water deficit conditions

It has been known that a variety of osmolytes which includes sugars (such as raffinose, trehalose, sucrose and sorbitol), sugar alcohols (such as mannitol), amino acids (such as proline), and secondary metabolites (such as isoflavonoids and saponins) will accumulate in the plants when it responses to the abiotic stress, [33–35]. These metabolism products are able to stabilize proteins and cellular structures to maintain cell turgor by osmotic adjustment. Redox metabolism removes excess levels of ROS (Reactive oxygen species) and re-establishes the cellular redox balance [36, 37]. The type of metabolism and related genes involved in the adjustment of soybean roots under water deficit conditions are still unclear. Galactinol and raffinose act as osmoprotectants and provide an adaptation to the water stress conditions [38]. Arabidopsis plants over-expressing GolS1 and GolS2 (galactinol synthase) accumulated high levels of galactinol and raffinose and were tolerant to drought and salinity stress [38, 39]. Several GOLS1 and GOLS2 genes that were up-regulated under the VMS, MS, and SS conditions were inhibited at the water-recovery phase in the present study. These candidate genes will be very promising and can be used for soybean transgenic development to improve soybean tolerance to water deficit conditions.

Starch serves as a primary carbohydrate source and can be mobilized to soluble sugars if the plants experience an environmental challenge. It is reported that the starch content will be depleted by drought and the soluble sugars will accumulate in the leaves by enhanced beta-amylase enzyme activity [40, 41]. Here, we found the reverse adjustment for the starch metabolism in the soybean root, which indicates that metabolites involved with energy production and growth were increased or shifted from shoots to roots. The total metabolism is accelerated to adapt to the water-deficit environment when water is a limiting factor. It is reported that the shoots and roots respond to drought in an opposite metabolic way, while shoots decrease their growth metabolism (lower concentrations of sugars, amino acids, nucleosides, nitrogen, phosphorus and potassium) and roots increase metabolism in a mirrored response [42]. BMY8 (β-amylase) antisense Arabidopsis plants accumulated high starch levels during cold shock (4 °C for 6 h) [43]. Three BMY8 genes were also found down-regulated under the MS and SS conditions in the soybean root, which indicates the GmBMY8 gene probably plays a role under drought stress by modulating starch metabolism.

As important membrane components, the changes in the composition of lipids may help to maintain membrane integrity and preserve cell compartmentation under water stress conditions. In response to drought, the total leaf lipid content decreased progressively. The time-course of the decrease in Arabidopsis leaf lipid content correlated well with the expression of genes involved in lipid degradation [44]. Most of the lipid biosynthesis related genes were down-regulated in the root in this study. In transgenic Arabidopsis with suppression of the inositol polyphosphate 1-phosphatase (IPP) signal by heterologous expression of the human type I inositol polyphosphate 5-phosphatase exhibited an increased drought tolerance phenotype, which was mediated in part via a DREB2A-dependent pathway [45]. The content and composition may change when plants are exposed to different abiotic and biotic stresses [46]. Little is known about the effects of water-deficit on lignin synthesis in roots until now. Research has shown that there was an increased expression of genes involved in lignin biosynthesis during the intermediate and final stages of water stress (from 48 h to 72 h) in rice roots (*Oryza sativa* L.), such as those coding for PAL, C3H, 4-coumarate: coenzyme A ligase (4CL), caffeoyl coenzyme A O-methyltransferase (CCoAOMT), cin-namyl alcohol dehydrogenase (CAD), and peroxidase [47]. Similarly, lignin synthesis-related proteins were induced mostly in the later stage of drought stress in the roots of wild watermelon (*Citrullus lanatus* sp.), which may function in the enhancement of physical desiccation tolerance and drought adaptation [48]. These results indicated that growth reduction and tolerance to water-deficit were associated with more lignin production in the roots. However, most of the lignin biosynthesis related genes in the soybean root showed a down-regulated pattern under the MS and SS conditions. The reason for this phenomenon may be caused by different regions of root and stress levels or degrees.

The analysis of metabolism related genes contributes to the understanding of soybean stress biology. Genetic engineering using genes encoding components of drought stress-related metabolic pathways has shown the potential to enhance drought resistance in soybeans [49–51]. Integration of proteomics and metabolomics analysis will provide more evidence to elucidate the nature of soybean water deficit stress responses in the future.

### Hormone interaction involved in the primary root development under water-deficit

Three hormones,auxin, ethylene and ABA, were identified to be involved in drought responsive pathways in soybean [52]. In this study, ethylene and auxin synthesis and signal transduction had more important roles than other hormones in the soybean taproot response to water deficient stress. At present, hormone interactions involved in abiotic stress were mainly derived from *Arabidopsis thaliana*. Until now, it was unknown how the hormone crosstalk regulated primary root elongation under water-deficit stress in soybean roots. Several genes (i.e., *GA2OX1*, *GA2OX2*, *ACS*, *CCD7*, *CCD8*, and *JAR1*) involved in different hormone crosstalk and related TFs were found in this study (Additional files 4, 5, 6 and 7), which indicated that the interactions between different hormones play a crucial role in soybean root development.

The expression of genes associated with the auxin transporter has been shown to be regulated by ethylene [53]. Conversely, auxin was found to affect the synthesis of ethylene [54]. Here, the expression of *PIN2* and *PIN5* were found to be regulated by the water-deficit stress, and several *ACS* genes (*ACS1*, *ACS6*, *ACS7*, *ACS8*, and *ACS10*) were regulated under MS and SS conditions. *CCD7* and *CCD8* genes were involved in the ABA and strigolactones (SL) synthesis pathway. SL, as plant hormone, functions as a positive regulator of primary root elongation and as a negative regulator of adventitious root formation due to modulation of auxin flux in the root [55–57]. Wang et al. reported that ABA treatment increased soybean *CCD7* and *CCD8* transcription [58]. Based on this study, hormone balance between SL and ABA might play an important role in primary root development under MS and SS treatments. Under drought stress, higher levels of ABA may up-regulate the activities of downstream TFs (MYBs), which would modify the auxin biosynthesis and trigger auxin responsive cascades, controlling root development. The increase in ABA content and signal transduction promotes the synthesis of ethylene, then inhibits the elongation of root. This complex network of hormone crosstalk allows cell differentiation to balance cell division and the formation of the root system architecture.

All of the above results give clues about transcripts associated with hormones that modulate root growth under drought stress. While progress has been made in identifying how water deficit affects hormone biosynthesis, response, and transport, the correlation between the content of hormones in the soybean taproot and the expression levels of genes are not well understood. It will be interesting and useful to investigate changes in hormone content in soybean taproots under water-deficit conditions in the future.

### Water-deficits induced TFs in soybean root

TFs are important classes of genes that regulate expression of downstream drought-responsive genes. In this study, several families, which include MYB, AP2, bHLH, WRKY, and Cys2His2 zinc-finger, were identified to be involved in soybean water-deficit responses either from tissue specific or from stress specific TFs. Among these TFs, MYB, MYC, AP2, and HD-ZIP families play a central role in drought tolerance [59–62]. These results predicated that soybean probably shares a conserved regulation mechanism with other species.

TFs DREB1A and DREB1D were found to be highly up-regulated by MS and SS conditions in the root. In transgenic Arabidopsis, overexpression of the DREB1/CBF family genes showed increasing tolerance to freezing, drought, and high salinity [62–64]. Overexpression of DREB/CBF TFs has been reported to enhance drought tolerance in soybeans [65, 66]. Drought is often accompanied by elevated air and leaf temperatures in the field. Heat shock TF (HSF) genes have been implicated not only in thermotolerance but also in plant growth and development and response to various stresses [67]. Six HSF transcription factors were found highly induced under the MS and SS conditions in soybean roots. After water recovery, the expression level decreased compared with SS treatment (Additional file 9). Several auxin, salt, and cold pathway related TFs were found in the DEG group in the root tissue. ICE1 encodes an upstream bHLH TF that regulates the transcription of CBF genes in the cold [68]. One soybean ICE TF (Glyma06g17330.1) was induced by the MS and SS treatments. Two auxin response TFs were affected; IAA26 (Glyma07g01800.1) was up-regulated and IAA19 (Glyma13g43780.1) was down-regulated by the stress. The induced IAA 26 in rice leaves could dynamically regulate the phytohormone IAA level to respond to drought stress [69]. Two PIF3 (phytochrome-interacting factor) genes (Glyma19g40980.1 and Glyma10g28290.1) were induced by the MS and SS treatments in the root. It was reported that the rice PIF-like protein Os-PIL1/PIL13 was a key regulator of reduced internode elongation in rice under drought conditions [70]. Overexpressing the STO (salt tolerance) gene in Arabidopsis conferred a higher salt tolerance phenotype than the wildtype through binding to a MYB TF [71]. One soybean STO (Glyma11g13570.1) gene was found induced under the MS and SS conditions, but was down-regulated after water recovery. Two GA pathway TFs; SCARECROW (SCR) and RGA-LIKE 2 della protein (RGL2), were also found to be down-regulated in all levels of drought stress. SCR is known to be a key regulator of stem cell renewal and radial patterning in the root of Arabidopsis [72, 73].

These results indicate that the water-deficit response in soybean roots may be involved in many different abiotic and hormone signaling pathways that modify the root growth. Further molecular work needs to be conducted to evaluate the importance of these TFs in the water-deficit response and to determine the role of individual genes. Investigating the transcriptional regulatory network of DEGs involved in different tissues at different water deficit stress levels would provide more information for future functional analysis.

## Conclusions

In conclusion, this study provides a large-scale transcriptome analysis of soybean taproot, leaf, and shoot in response to different water-deficit levels and demonstrated the usefulness of the DEG approach for identifying key genes across tissues and stress levels. The DEGs involved in metabolism, hormone, and regulation networks were identified and proved to be associated with the development of soybean roots under drought. Our gene expression analysis results not only provided detailed and accurate information for characterizing the drought stress response in soybeans but also provided candidates for genetic engineering for stress resistance in soybeans. Genetic modifications of these candidates could play a vital role in achieving drought tolerance and yield performance.

## Methods

### Plant growth conditions and stress treatment

Williams 82 soybean seeds were planted in 5-gallon pots filled with soil media (turface and sand mixed in 2:1 ratio) in the growth chamber at the Sears Plant Growth Facility at the University of Missouri. The controlled conditions of the growth chamber was 27/21 °C day/night temperature, photoperiod of 16/8 day/night, 60 % relative humidity. Three seeds were sown per pot and thinned to one plant per pot when the plants had two sets of unfolded trifoliate leaves (Vegetative (V) 2 stage). All pots were kept well-watered until V3 (three unfolded trifoliate leaves) stage [74]. For very mild stress (VMS), drought stress was imposed by withholding water for 5 days. For mild stress (MS), drought stress was imposed by withholding water for 12 days. For severe stress (SS), drought stress was imposed by withholding water for 19 days. For water recovery after severe stress (SR), plants were re-watered for 2 days after withholding water for 19 days. Each treatment had corresponding control plants and all control plants were kept well-watered until sampling. The soil moisture of each pot was measured on a daily basis to monitor the treatment condition by using the PR2 soil moisture profile probe (Delta-T Devices Ltd., UK). The value recorded at the sampling day was used for Fig. 1b. Plant height, water potential, stomatal conductance, canopy temperature, and leaf relative water content (RWC) were recorded or determined at the sampling day in all control and stressed plants. These physiological traits were collected from an attached leaflet of the fourth trifoliate leaf from the main-stem apex at midday. Full leaves were used in the determination of RWC. Water potential was measured using a pressure chamber (PMS Instrument Co., Albany, CA, USA). A LI-COR 6400XT portable photosynthesis system (LI-COR, Lincoln, NE) was used to measure net stomatal conductance. Canopy temperatures were collected with an infrared meter (FLIP Ex severe infra-red meter, Everett, WA). RWC was determined by the ratio of tissue fresh weight to tissue turgid weight. This is termed relative tissue weight.

Five-centimeter long root tissues from the root tip, the first fully expanded trifoliate, and the entire shoot bearing third trifoliate leaf were collected along with their respective controls. Three individual plants were used for tissue sampling, measurement, and RNA isolation. Three independent biological replicates were used to reduce technical and biological variability. The collected tissues were rinsed with RNase-free water immediately to remove sand and turface, and then blotted to remove excess water. Each samples were wrapped with aluminum foil, and flash frozen in liquid nitrogen and stored in a -80 °C freezer for further RNA isolation.

### RNA isolation, library construction, and RNA sequencing

Total RNA was isolated from the roots with the RNeasy Plant mini kit (Qiagen, Cat#:74904) following the manufacturer’s protocols. On-column DNase digestion with the RNase-Free DNase set were used to remove the DNA contamination (Qiagen, Valencia, CA, Cat#:79254). The RNA quantity was checked with a NanoDrop Spectrometer (ND-1000 Spectrophotometer, Thermo Scientific). The RNA integrity was analyzed using the Agilent 2100 Bioanalyzer (RNA Nano Chip, Agilent, Santa Clara, CA, USA). The RNA integrity number value ≥7 was used to select the best RNA for library construction. Total RNA, 2 μg from each sample, was used for library construction. The RNA TruSeq Stranded mRNA LT sample prep kit (Illumina, San Diego, CA, RS-122-2101) was used to prepare the RNA-Seq library following the manufacturer’s protocols. A Qubit quantitation assay and the NGS Fragment Analysis (Agilent 2100 Bioanalyzer, Santa Clara, CA) were conducted on each library. Single end reads were generated by the Illumina HiSeq 2000 (read length 1 × 100 base; Illumina, Inc. San Diego, CA).

### Mapping of RNA-Seq reads

The initial base calling and quality filtering of the reads generated with the Illumina analysis pipeline (Fastq format) were performed using a custom Perl script and the default parameters of the Illumina pipeline (http://www.illumina.com). Additional filtering for poor-quality bases was performed using the FASTX-toolkit available in the FastQC software package (http://www.bioinformatics.babraham.ac.uk/projects/fastqc/). High-quality mRNA-Seq reads were aligned to the *Glycine max* reference genome (Gmax1.1 version) and Phytozome v9.0 gene model release using Tophat (version 140 1.4.1) [75]. The genome indexes for Tophat were built using the Bowtie build command of Bowtie (version 0.12.7) with the reference genome file as input [76]. Tophat was then run with default parameters and a reference GTF file using the –G option, and replicates of each treatment/sample were mapped independently to improve alignment sensitivity and accuracy for further analysis.

### Sequence data analysis and differential counting

Only uniquely mapped reads were used in the analysis. The gene expression (FPKM) levels were estimated using Cufflinks software (version v2.1.1) [77] while differential gene expression analysis was performed using Cuffdiff (version v2.1.1) among the different sample comparisons. Only the genes with more than a 2-fold change and a *p*-value less than 5*10^−5^ were considered as significant differentially expressed genes. We used the R value and the absolute value of log2 ratio > 2 as the threshold to judge the significance of gene expression differences. The analyzed RNA-Seq gene expressions and the differential gene expression results are available for browsing in Soybean Knowledge Base (SoyKB) [78] at http://soykb.org. The datasets (SoyKB/Information/Dataset 17) can be browsed using the differential expression analysis suite tools, which provide access to the gene lists, Venn diagrams, Volcano plots, and function for pathway analysis.

### Pathway identification and TF analysis

The different levels of water-deficit regulated genes were clustered using dChip software and the response pathways were plotted by MapMan [79]. Multiple biological or metabolic pathways were plotted together with the mapped gene intensity of fold change by a green and red schema. The statistical cutoff of the fold change was two.

### Promoter analysis

Promoter sequences (2,000 bp upstream) of the translation site of DEGs were extracted from the Phytozome database (http://www.phytozome.net/). The presence and abundance of the known *cis*-elements were analyzed using the DREAM tool [80] in MEME (based on E value <0.05). Then, similar motif searches were made from the JASPAR CORE (2014) plants database in Tomtom tool [81] (another MEME suite program).

### Quantitative real-time PCR analysis

Quantitative real-time PCR was performed on an ABI7900HT detection system (Life Technologies, Foster City, CA) using the Maxima SYBR Green/ROX qPCR Master Mix (2×) (Cat# K0223, Thermo, USA) following the manufacturer’s protocol. PCR amplification was achieved by the following program: 50 °C for 2 min, 95 °C for 10 min, and then 40 cycles of 95 °C for 15 s, 60 °C for 1 min. The comparative Ct method for quantification was used to quantify the relative expression of specific genes [82]. Actin (Glyma18g52780) was selected as an internal control to normalize gene expression. All primers were designed using Primer3 web-interface (http://frodo.wi.mit.edu/primer3/input.htm; [83]). The primer sequences are listed in Additional file 13. The reactions were performed with three biological replicates and repeated once for technical replicate.

### Availability of supporting data

The data sets supporting the results of this article are included within the article (and its additional files). Illumina sequences were deposited to the NCBI Sequence Read Archive under the accessions SRP067593 (http://www.ncbi.nlm.nih.gov/sra/SRP067593).

## Additional files

Additional file 1:
**Gene annotation and expression analysis for primary metabolism.** (XLSX 26 kb) Additional file 2:
**Gene annotation and expression analysis for secondary metabolism.** (XLSX 19 kb) Additional file 3:
**Gene annotation and expression analysis for carbohydrate metabolism.** (XLSX 12 kb) Additional file 4:
**Gene annotation and expression analysis of auxin synthesis and the response pathway.** (XLSX 11 kb) Additional file 5:
**Gene annotation and expression analysis of ethylene synthesis and the response pathway.** (XLSX 15 kb) Additional file 6:
**Gene annotation and expression analysis of ABA synthesis and the response pathway.** (XLSX 10 kb) Additional file 7:
**Gene annotation and expression analysis of JA, GA, and SA synthesis and the response pathway.** (XLSX 13 kb) Additional file 8:
**The regulated transcription factor (TFs) number list for different tissues and water-deficit treatments.** Total number of TFs in different tissues, tissue-specific TFs under all stress treatments, total number of TFs under different stress treatments, and stress specific TFs in all tissues are also presented. (XLSX 9 kb) Additional file 9:
**The gene list of changed transcription factors in roots under MS and SS treatments.** (XLSX 18 kb) Additional file 10:
**The gene list and expression patterns for all transcription factors involved in all tissues and stress treatments.** (XLSX 12 kb) Additional file 11:
**Enriched motif analysis in each group involved in roots and different stress levels.** The DREAM tool was used to reveal over-represented elements in the promoter regions of certain groups of genes that were differentially regulated in roots and different stress levels. (XLSX 16 kb) Additional file 12:
**High correlation between RNA-Seq data (X axis) and qRT-PCR data (y axis) using the log2 values for different stress levels.** (PPTX 38 kb) Additional file 13:
**Gene information used for qRT-PCR verification of RNA-Seq data and corresponding expression patterns extracted by RNA-Seq.** (XLSX 10 kb)

## Abbreviations

ABA: abscisic acid
ACS: ACC synthase
AP2: activating Protein 2
BES: BRI1-EMS-SUPPRESSOR
bHLH: basic helix-loop-helix
CCD7: carotenoid cleavage dioxygenases 7
CCD8: carotenoid cleavage dioxygenases 8
DEGs: differentially expressed genes
ERFs: ethylene response factors
FA: fatty acid
GA: gibberellin
GA2OX1: gibberellin 2-oxidase 1
GmJAR1: JASMONATE RESISTANT 1
HSF: Heat shock TF
JA: jasmonic acid
LWP: leaf water potential
MPa: megapascals
MS: mild stress
MSMC: Missouri Soybean Merchandising Council
MYB: myeloblastosis
NCED: 9-cis-epoxycarotenoid dioxygenase
NGS: next-generation sequencing
PIF3: phytochrome-interacting factor
PMEs: pectin methylesterases
RGL2: RGA-LIKE 2 della protein
RWC: relative water content
SA: salicylic acid
SCR: SCARECROW
SL: strigolactones
SS: severe stress
STO: salt tolerance
TFs: transcription factors
VMS: very mild stress
VPD: high vapor pressure deficit
WRKY: a class of DNA-binding proteins that recognize the TTGAC(C/T) W-box elements
XTHs: xyloglucan endotransglucosylase/hydrolases

## References

[1] Stacey G, Vodkin L, Parrott WA, Shoemaker RC (2004). National science foundation-sponsored workshop report. Draft plan for soybean genomics. Plant Physiol.

[2] Sprent JI (2001). Nodulation in legumes.

[3] Dogan E, Kirnak H, Copur O (2007). Deficit irrigations during soybean reproductive stages and CROPGRO-soybean simulations under semi-arid climatic treatments. Field Crop Res.

[4] Yamaguchi M, Sharp RE (2010). Complexity and coordination of root growth at low water potentials: recent advances from transcriptomic and proteomic analyses. Plant Cell Environ.

[5] Lenssen A. Soybean response to drought. Integrated Crop Management News Online. Iowa State University Extension. 2012. http://crops.extension.iastate.edu/cropnews/2012/06/soybean-response-drought. Accessed 22 Jun 2012.

[6] Hirasawa T, Tanaka K, Miyamoto D, Takei M, Ishihara K (1994). Effects of pre-flowering moisture deficits on dry matter production and ecophysiological characteristics in soybean plants under drought treatments during grain filling. Jpn J Crop Sci.

[7] Hoogenboom G, Peterson CM, Huck MG (1987). Shoot growth rate of soybeans as affected by drought stress. Agron J.

[8] Taylor HM, Burnett E, Booth GD. Taproot elongation rates of soybeans. Z. Acker Pflanzenbau Bd. 1978;146:33.

[9] Wang G, Zhu Q, Meng Q, Wu C (2012). Transcript profiling during salt stress of young cotton (Gossypium hirsutum) seedlings via Solexa sequencing. Acta Physiol Plant.

[10] Yu S, Zhang F, Yu Y, Zhang D, Zhao X, Wang WH (2012). Transcriptome profiling of dehydration stress in the Chinese cabbage (Brassica rapa L. ssp. pekinensis) by tag sequencing. Plant Mol Biol Rep.

[11] Wang X, Liu Y, Jia Y, Gu H, Ma H, Yu T (2012). Transcriptional responses to drought stress in root and leaf of chickpea seedling. Mol Biol Rep.

[12] Shen Y, Zhang Y, Chen J, Lin H, Zhao M, Peng H (2013). Genome expression profile analysis reveals important transcripts in maize roots responding to the stress of heavy metal Pb. Physiol Plant.

[13] Zhou Y, Gao F, Liu R, Feng J, Li H (2012). De novo sequencing and analysis of root transcriptome using 454 pyrosequencing to discover putative genes associated with drought tolerance in Ammopiptanthus mongolicus. BMC Genomics.

[14] Fan XD, Wang JQ, Yang N, Dong YY, Liu L, Wang FW (2013). Gene expression profiling of soybean leaves and roots under salt, saline-alkali and drought stress by high-throughput Illumina sequencing. Gene.

[15] Shin JH, Vaughn JN, Abdel-Haleem H, Chavarro C, Abernathy B, Kim KD (2015). Transcriptomic changes due to water deficit define a general soybean response and accession-specific pathways for drought avoidance. BMC Plant Biol.

[16] Prince SJ, Joshi T, Mutava RN, Syed N, Joao Vitor Mdos S, Patil G (2015). Comparative analysis of the drought-responsive transcriptome in soybean lines contrasting for canopy wilting. Plant Sci.

[17] Devi MJ, Sinclair TR, Taliercio E (2015). Comparisons of the effects of elevated vapor pressure deficit on gene expression in leaves among two fast-wilting and a slow-wilting soybean. PLoS One.

[18] Ha CV, Watanabe Y, Tran UT, Le DT, Tanaka M, Nguyen KH (2015). Comparative analysis of root transcriptomes from two contrasting drought-responsive Williams 82 and DT2008 soybean cultivars under normal and dehydration conditions. Front Plant Sci.

[19] Seki M, Umezawa T, Urano K, Shinozaki K (2007). Regulatory metabolic networks in drought stress responses. Curr Opin Plant Biol.

[20] Peleg Z, Blumwald E (2011). Hormone balance and abiotic stress tolerance in crop plants. Curr Opin Plant Biol.

[21] Xu CC, Zou Q (1993). Effect of drought on lipoxygenase activity, ethylene, and ethane formation in leaves of soybean plants. Acta Bot Sin.

[22] Morgan PW, Drew MC (1997). Ethylene and plant responses to stress. Physiol Plant.

[23] Schramm F, Larkindale J, Kiehlmann E, Ganguli A, Englich G, Vierling E (2008). A cascade of transcription factor DREB2A and heat stress transcription factor HsfA3 regulates the heat stress response of Arabidopsis. Plant J.

[24] Ding Z, Li S, An X, Liu X, Qin H, Wang D (2009). Transgenic expression of MYB15 confers enhanced sensitivity to abscisic acid and improved drought tolerance in Arabidopsis thaliana. J Genet Genomics.

[25] Do JH, Choi DK (2008). Clustering approaches to identifying gene expression patterns from DNA microarray data. Mol Cells.

[26] Tavazoie S, Hughes JD, Campbell MJ, Cho RJ, Church GM (1999). Systematic determination of genetic network architecture. Nat Genet.

[27] Ramireddy E, Chang L, Schmülling T (2014). Cytokinin as a mediator for regulating root system architecture in response to environmental cues. Plant Signal Behav.

[28] Klucher KM, Chow H, Reiser L, Fischer RL (1996). The AINTEGUMENTA gene of Arabidopsis required for ovule and female gametophyte development is related to the floral homeotic gene APETALA2. Plant Cell.

[29] Ma J, Li MY, Wang F, Tang J, Xiong AS (2015). Genome-wide analysis of Dof family transcription factors and their responses to abiotic stresses in Chinese cabbage. BMC Genomics.

[30] He L, Su C, Wang Y, Wei Z. ATDOF5.8 protein is the upstream regulator of ANAC069 and is responsive to abiotic stress. Biochimie. 2015. doi: 10.1016/j.biochi.2014.12.017. 10.1016/j.biochi.2014.12.017 25572919

[31] Zhang Y, Zhao L, Li H, Gao Y, Li Y, Wu X (2013). GmGBP1, a homolog of human ski interacting protein in soybean, regulates flowering and stress tolerance in Arabidopsis. BMC Plant Biol.

[32] Spollen WG, Sharp RE, Saab IN, Wu Y, Smith JAC, Griffiths H (1993). Regulation of cell expansion in roots and shoots at low water potentials. Water deficits, plant responses from cell to community.

[33] Urano K, Kurihara Y, Seki M, Shinozaki K (2010). ‘Omics’ analyses of regulatory networks in plant abiotic stress responses. Curr Opin Plant Biol.

[34] Wu W, Zhang Q, Zhu Y, Lam HM, Cai Z, Guo D (2008). Comparative metabolic profiling reveals secondary metabolites correlated with soybean salt tolerance. J Agric Food Chem.

[35] Yamaguchi M, Valliyodan B, Zhang J, Lenoble ME, Yu O, Rogers EE (2010). Regulation of growth response to water stress in the soybean primary root. I. Proteomic analysis reveals region-specific regulation of phenylpropanoid metabolism and control of free iron in the elongation zone.. Plant Cell Environ.

[36] Gábor K, Robert L, Gabriella S, Virág S, Lívia SS, Gábor G (2005). Genetic manipulation of proline levels affects antioxidants in soybean subjected to simultaneous drought and heat stresses. Physiol Plant.

[37] Valliyodan B, Nguyen HT (2006). Understanding regulatory networks and engineering for enhanced drought tolerance in plants. Curr Opin Plant Biol.

[38] Taji T, Ohsumi C, Iuchi S, Seki M, Kasuga M, Kobayashi M (2002). Important roles of drought- and cold-inducible genes for galactinol synthase in stress tolerance in Arabidopsis thaliana. Plant J.

[39] Nishizawa A, Yabuta Y, Shigeoka S (2008). Galactinol and raffinose constitute a novel function to protect plants from oxidative damage. Plant Physiol.

[40] Todaka D, Matsushima H, Morohashi Y (2000). Water stress enhances beta-amylase activity in cucumber cotyledons. J Exp Bot.

[41] Kaplan F, Guy CL (2004). beta-Amylase induction and the protective role of maltose during temperature shock. Plant Physiol.

[42] Gargallo-Garriga A, Sardans J, Pérez-Trujillo M, Rivas-Ubach A, Oravec M, Vecerova K (2014). Opposite metabolic responses of shoots and roots to drought. Sci Rep.

[43] Kaplan F, Guy CL (2005). RNA interference of Arabidopsis beta-amylase8 prevents maltose accumulation upon cold shock and increases sensitivity of PSII photochemical efficiency to freezing stress. Plant J.

[44] Gigon A, Matos AR, Laffray D, Zuily-Fodil Y, Pham-Thi AT (2004). Effect of drought stress on lipid metabolism in the leaves of Arabidopsis thaliana (ecotype Columbia). Ann Bot.

[45] Perera IY, Hung CY, Moore CD, Stevenson-Paulik J, Boss WF (2008). Transgenic Arabidopsis plants expressing the type 1 inositol 5-phosphatase exhibit increased drought tolerance and altered abscisic acid signaling. Plant Cell.

[46] Moura JC, Bonine CA, de Oliveira Fernandes Viana J, Dornelas MC, Mazzafera P (2010). Abiotic and biotic stresses and changes in the lignin content and composition in plants. J Integr Plant Biol.

[47] Yang L, Wang CC, Guo WD, Li XB, Lu M, Yu CL (2006). Differential expression of cell wall related genes in the elongation zone of riceroots under water deficit. Russ J Plant Physiol.

[48] Yoshimura K, Masuda A, Kuwano M, Yokota A, Akashi K (2008). Programmed proteome response for drought avoidance/tolerancein the root of a C-3 xerophyte (wild watermelon) under water deficits. Plant Cell Physiol.

[49] de Ronde JA, Cress WA, Krüger GH, Strasser RJ, Van Staden J (2004). Photosynthetic response of transgenic soybean plants, containing an Arabidopsis P5CR gene, during heat and drought stress. J Plant Physiol.

[50] de Ronde JA, Laurie RN, Caetano T, Greyling MM, Kerepesi I (2004). Comparative study between transgenic and non-transgenic soybean lines proved transgenic lines to be more drought tolerant. Euphytica.

[51] Simon-Sarkadi L, Kocsy G, Várhegyi A, Galiba G, de Ronde JA (2005). Genetic manipulation of proline accumulation influences the concentrations of other amino acids in soybean subjected to simultaneous drought and heat stress. J Agric Food Chem.

[52] Le DT, Nishiyama R, Watanabe Y, Tanaka M, Seki M, Ham le H, et al. Differential gene expression in soybean leaf tissues at late developmental stages under drought stress revealed by genome-wide transcriptome analysis. PLoS One. 2012. doi: 10.1371/journal.pone.0049522. 10.1371/journal.pone.0049522 PMC3505142 23189148

[53] Růzicka K, Ljung K, Vanneste S, Podhorská R, Beeckman T, Friml J (2007). Ethylene regulates root growth through effects on auxin biosynthesis and transport-dependent auxin distribution. Plant Cell.

[54] Tsuchisaka A, Theologis A (2004). Unique and overlapping expression patterns among the Arabidopsis 1-amino-cyclopropane-1-carboxylate synthase gene family members. Plant Physiol.

[55] Ruyter-Spira C, Kohlen W, Charnikhova T, van Zeijl A, van Bezouwen L, de Ruijter N (2011). Physiological effects of the synthetic strigolactone analog GR24 on root system architecture in Arabidopsis: another belowground role for strigolactones?. Plant Physiol.

[56] Rasmussen A, Mason MG, De Cuyper C (2012). Strigolactones suppress adventitious rooting in Arabidopsis and pea. Plant Physiol.

[57] Koltai H (2011). Strigolactones are regulators of root development. New Phytol.

[58] Wang RK, Wang CE, Fei YY, Gai JY, Zhao TJ (2013). Genome-wide identification and transcription analysis of soybean carotenoid oxygenase genes during abiotic stress treatments. Mol Biol Rep.

[59] Shin DJ, Moon SJ, Han S, Kim BG, Park SR, Lee SK (2011). Expression of StMYB1R-1, a novel potato single MYB-like domain transcription factor increases drought tolerance. Plant Physiol.

[60] Sugano S, Kaminaka H, Rybka Z, Catala R, Salinas J, Matsui K (2003). Stress-responsive zinc finger gene ZPT2-3 plays a role in drought tolerance in petunia. Plant J.

[61] Yu H, Chen X, Hong YY, Wang Y, Xu P, Ke SD (2008). Activated expression of an Arabidopsis HD-START protein confers drought tolerance with improved root system and reduced stomatal density. Plant Cell.

[62] Liu Q, Kasuga M, Sakuma Y, Abe H, Miura S, Yamaguchi-Shino-zaki K (1998). Two transcription factors, DREB1 and DREB2, with an EREBP/AP2 DNA binding domain separate two cellular signal transduction pathways in drought- and low-temperature-respon-sive gene expression, respectively, in Arabidopsis. Plant Cell.

[63] Jaglo-Ottosen KR (1998). Arabidopsis CBF1 overexpression induces COR genes and enhances freezing tolerance. Science.

[64] Kasuga M, Liu Q, Miura S, Yamaguchi-Shinozaki K, Shinozaki K (1999). Improving plant drought, salt, and freezing tolerance by gene transfer of a single stress-inducible transcription factor. Nat Biotechnol.

[65] Polizel AM, Medri ME, Nakashima K, Yamanaka N, Farias JR, de Oliveira MC (2011). Molecular, anatomical and physiological properties of a genetically modified soybean line transformed with rd29A:AtDREB1A for the improvement of drought tolerance. Genet Mol Res.

[66] de Paiva Rolla AA, de Fátima Corrêa Carvalho J, Fuganti-Pagliarini R, Engels C, do Rio A, Marin SR (2014). Phenotyping soybean plants transformed with rd29A:AtDREB1A for drought tolerance in the greenhouse and field. Transgenic Res.

[67] Nishizawa A, Yabuta Y, Yoshida E, Maruta T, Yoshimura K, Shigeoka S (2006). Arabidopsis heat shock transcription factor A2 as a key regulator in response to several types of environmental stress. Plant J.

[68] Chinnusamy V, Ohta M, Kanrar S, Lee BH, Hong X, Agarwal M (2003). ICE1: a regulator of cold-induced transcriptome and freezing tolerance in Arabidopsis. Genes Dev.

[69] Wang D, Pan YJ, Zhao XQ, Zhu LH, Fu BY, Li ZK (2011). Genome-wide temporal-spatial gene expression profiling of drought responsiveness in rice. BMC Genomics.

[70] Todaka D, Nakashima K, Maruyama K, Kidokoro S, Osakabe Y, Ito Y (2012). Rice phytochrome-interacting factor-like protein OsPIL1 functions as a key regulator of internode elongation and induces a morphological response to drought stress. Proc Natl Acad Sci U S A.

[71] Nagaoka S, Takano T (2003). Salt tolerance‐related protein STO binds to a Myb transcription factor homologue and confers salt tolerance in Arabidopsis. J Exp Bot.

[72] Di Laurenzio L, Wysocka-Diller J, Malamy JE, Pysh L, Helariutta Y, Freshour G (1996). The SCARECROW gene regulates an asymmetric cell division that is essential for generating the radial organization of the Arabidopsis root. Cell.

[73] Helariutta Y, Fukaki H, Wysocka-Diller J, Nakajima K, Jung J, Sena G (2000). The SHORT-ROOT gene controls radial patterning of the Arabidopsis root through radial signaling. Cell.

[74] Fehr WR, Caviness CE, Burmood DT, Pennington JS (1971). Stage of development descriptions for soybeans, Glycine max (L.) Merr. Crop Sci.

[75] Trapnell C, Pachter L, Salzberg SL (2009). TopHat: discovering splice junctions with RNA-Seq. Bioinformatics.

[76] Langmead B, Salzberg SL (2012). Fast gapped-read alignment with Bowtie 2. Nat Methods.

[77] Trapnell C, Hendrickson DG, Sauvageau M, Goff L, Rinn JL, Pachter L (2013). Differential analysis of gene regulation at transcript resolution with RNA-seq. Nat Biotechnol.

[78] Joshi T, Fitzpatrick MR, Chen S, Liu Y, Zhang H, Endacott RZ (2014). Soybean knowledge base (SoyKB): a web resource for integration of soybean translational genomics and molecular breeding. Nucleic Acids Res.

[79] Usadel B, Poree F, Nagel A, Lohse M, Czedik-Eysenberg A, Stitt M (2009). A guide to using MapMan to visualize and compare Omics data in plants: a case study in the crop species, Maize. Plant Cell Environ.

[80] Bailey TL (2011). DREME: Motif discovery in transcription factor ChIP-seq data. Bioinformatics.

[81] Gupta S, Stamatoyannopoulos JA, Bailey TL, Noble WS (2007). Quantifying similarity between motifs. Genome Biol.

[82] Livak KJ, Schmittgen TD (2001). Analysis of relative gene expression data using real-time quantitative PCR and the 2-[Delta][Delta]CT method. Methods.

[83] Rozen S, Skaletsky H (2000). Primer3 on the WWW for general users and for biologist programmers. Methods Mol Biol.

