# Supplementary material for: Genome-wide transcriptome analysis of soybean primary root under varying water-deficit conditions
Source: BMC Genomics. 2016 Jan 15;17:57. doi: 10.1186/s12864-016-2378-y (PMC4714440; doi:10.1186/s12864-016-2378-y)
Supplement: Additional file 12: — High correlation between RNA-Seq data (X axis) and qRT-PCR data (y axis) using the log2 values for different stress levels. (PPTX 38 kb) [file 12864_2016_2378_MOESM12_ESM.pptx]

## Slide 1
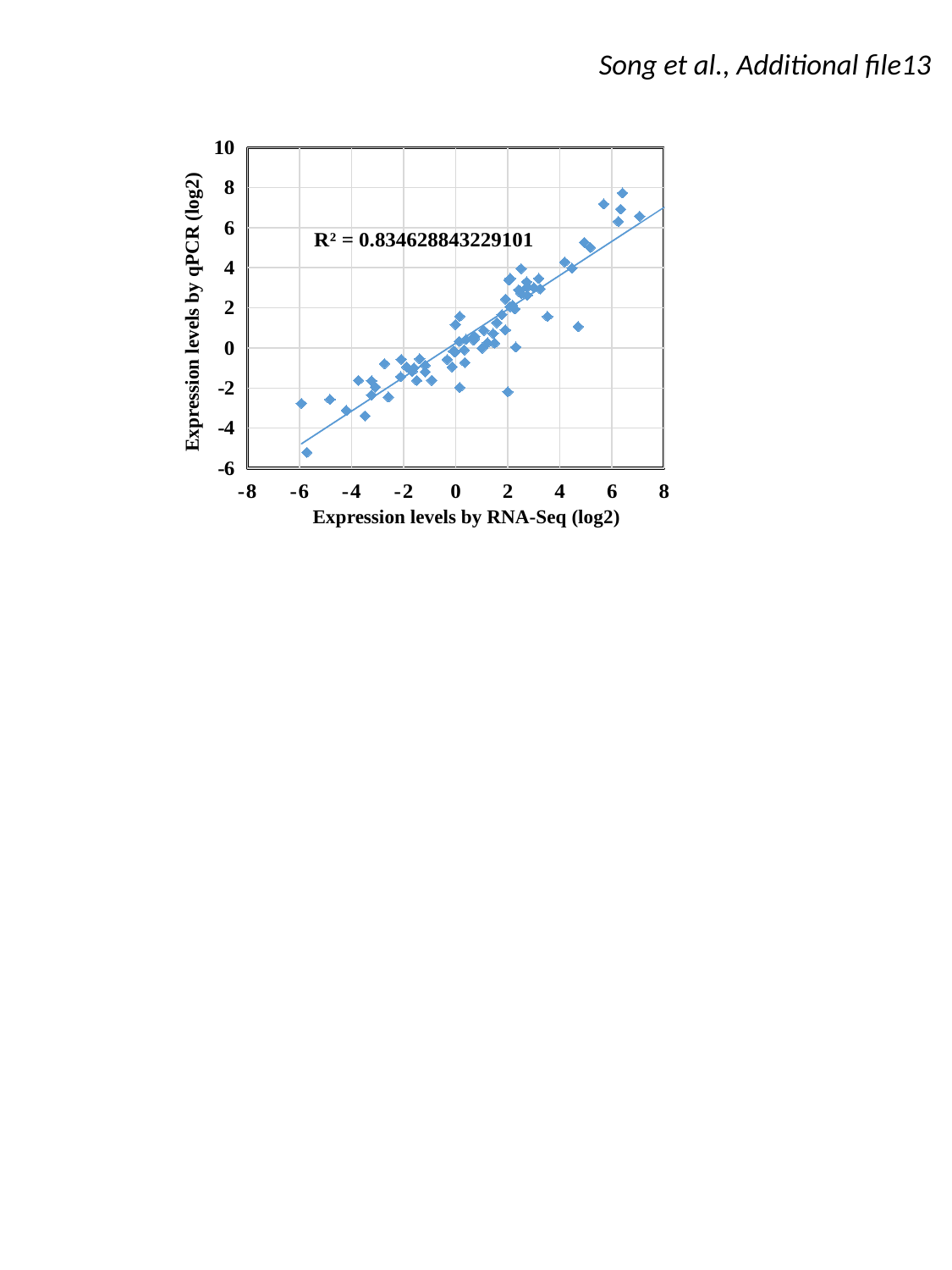

Song et al., Additional file13
### Chart
| Category | qPCR |
|---|---|Expression levels by qPCR (log2)
Expression levels by RNA-Seq (log2)
